# An Exploration of Nutritional Education within the Holiday Activities and Food Programme in England

**DOI:** 10.3390/ijerph19042398

**Published:** 2022-02-19

**Authors:** Emily K. Round, Jackie Shinwell, Paul B. Stretesky, Margaret Anne Defeyter

**Affiliations:** Healthy Living Lab, Faculty of Health & Life Sciences, Northumbria University, Newcastle-Upon-Tyne NE1 8ST, UK; j.shinwell@northumbria.ac.uk (J.S.); paul.stretesky@northumbria.ac.uk (P.B.S.)

**Keywords:** holiday hunger, nutritional education, holiday programme, food insecurity, holiday activities and food, HAF, school holidays, food literacy

## Abstract

Nutritional education is a recent, mandatory inclusion within the quality standards framework for the Holiday Activities and Food (HAF) programme in England; funded by the Department for Education (DfE). Whilst research has been conducted regarding nutritional education in other contexts, such as schools and community organisations, to the authors’ knowledge, no published research has yet explored nutritional education within HAF. The current study therefore aimed to explore the implementation, delivery, and perceived facilitators, barriers and impacts of nutritional education across a number of Local Authorities delivering HAF in England. Purposive sampling (*n* = 11) was used to recruit HAF leads involved in nutritional education, to participate in semi-structured interviews. Thematic analysis showed that nutritional education is currently delivered through a variety of modes including face-to-face, online, and take-home methods, all of which require a range of considerations in terms of implementation, delivery, and associated impacts, with some holiday clubs offering no nutritional education. According to participating HAF leads, nutritional education was used as a mechanism to enhance children’s and parents’ cooking confidence and competence, to improve dietary intake, and to increase understanding of issues such as food sustainability, environmental impacts, and food provenance. Although there are many examples of innovative practice, the findings suggested that COVID guidelines proved challenging for providers to include nutritional education within HAF delivery during 2021. Further, whilst the quality standards framework for nutritional education provides flexibility in terms of implementation and delivery, specific guidance, and monitoring of provision is required to ensure quality assurance and consistency across the HAF programme.

## 1. Introduction

During term-time, almost 20% of school-aged children in England are eligible for free school meals [1,2], with research by The Food Foundation demonstrating that approximately 2.5 million children experienced food insecurity in the six months up until July 2021. Problematically, however, without free school meal provision during school holiday periods, many families face increased financial pressures and are at further risk of food insecurity, known as holiday hunger [3,4]. To alleviate these issues, holiday clubs, which extend beyond solely providing access to food, have formed over recent years [5,6] and research has demonstrated significant social and health benefits of attendance, both for school-aged children and their wider families [7,8,9]; with food insecure households benefitting most from holiday provision [10]. Importantly, food diaries have demonstrated that attendance at holiday clubs improves dietary intake, with a higher consumption of core-foods on attendance days [7], and better adherence to UK dietary guidelines [11,12]. Moreover, both children and parents have reported numerous positive benefits of attending holiday clubs, including improved physical, social, and emotional wellbeing, and dietary intake [7,12].

In 2021, the Department for Education (DfE) announced that it would provide financial support to holiday clubs by contributing £220 M of funding to all 151 higher-tier Local Authorities in England, under a centralised scheme called Holiday Activities and Food (HAF). HAF aims to support families of children, who are in receipt of free school meals during term-time, when school is not in session. The DfE’s decision to invest in HAF was informed by several HAF pilots, and works by funding both existing and newly established holiday clubs through Local Authorities that distribute DfE financial support, and aid in coordinating provision and activities. The HAF programme provides support to families, through access to nutritious meals that adhere to School Food Standards, provides children with access to physical activities and enrichment opportunities, and refers parents to relevant support services (e.g., debt advice). Moreover, HAF programmes are required to include daily nutritional education sessions to children, and weekly training and advice for the wider family on sourcing, preparing, and cooking nutritious, low-cost food [13].

The inclusion of nutritional education in HAF is most likely based on prior research that suggests that community-based cooking programmes improve the dietary intake of both school-aged children and their wider families [14]. Such sessions have been shown to enhance cooking confidence and nutritional knowledge, which in turn, improves dietary habits and food literacy [15]. Other child-focused cooking programmes have similarly demonstrated benefits surrounding cooking competence and willingness to try new foods, which is assumed to relate to the repeated exposure to foods during the sessions and improved self-efficacy [16]. Likewise, food education is a compulsory element of the school curriculum in England, and research on school-based nutritional education has found a whole-school approach, involving a range of repeated social and practical food experiences such as group education on food provenance, cooking and tasting experiences, to be beneficial for cooking competence, wellbeing and dietary change [17,18]. Finally, the school-dining experience affords opportunities for social interactions with peers, which has shown to improve well-being and dietary habits, including trying of foods and dining etiquette, through social learning and modelling [19]; such environments may therefore play a vital role in increasing children’s food literacy and cooking confidence within the HAF programme.

Although nutritional education is a mandatory element of HAF, as outlined in the DfE quality standards framework, no published research has yet explored nutritional education within HAF. As all providers are required to follow DfE standards, exploring how nutritional education is implemented and delivered is important to service improvement and HAF programme evaluation. The DfE guidance states that nutritional education may be delivered at HAF programmes through a variety of methods, including practical demonstrations of food preparation, cooking, modelling food behaviours, and more formal nutritional education per se. As the DfE requires no robust monitoring of nutritional education, there is no record of how often this element of the HAF programme is being delivered, and how it is being delivered. Thus, the aims of the current study are to explore the implementation, delivery and perceived facilitators, barriers and impacts of nutritional education across a number of Local Authorities delivering HAF in England.

## 2. Materials and Methods

### 2.1. Study Design

A qualitative design was used for this study, conducting semi-structured interviews with HAF leads at the Local Authority or coordinator level of the programme. This method was considered appropriate as semi-structured interviews are often used within research obtaining the views of key stakeholders [20], and the aim of the study was to explore how the implementation of nutritional education was approached by Local Authorities.

### 2.2. Participants

Participants (*n* = 11) were recruited using non-probability purposive sampling. All participants were HAF leads who were actively involved in the implementation and delivery of nutritional education at HAF programmes from eight Local Authorities in England, and therefore had detailed knowledge at their respective Local Authority level. The Local Authorities were geographically spread from the south to the north of England. An invitation letter was distributed through the HAF Alliance, and Local Authorities who were interested in participating contacted the researcher via email. Demographic data were self-reported by participants. Ages ranged between 28–65 years old (Mean age = 42.9 years, SD = 10.4); nine participants identified as female, and two participants identified as male. Participants identified their ethnicity as White British (*n* = 7), Irish (*n* = 2), Indian (*n* = 1), and African (*n* = 1). All participants were fluent in English.

### 2.3. Procedure

Following the receipt of ethical approval (reference no: 33856) from the Faculty of Health and Life Sciences Research Ethics Committee at Northumbria University, information about the study was distributed through the HAF Alliance [21]—a collaboration of charities and organisations involved with the implementation and delivery of HAF programmes in England. Staff leading the provision of HAF with, or for, Local Authorities who were interested in participating in the study were asked to contact the research team and were then sent a letter of invitation, participant information sheets, and consent forms. Following the receipt of signed informed consent forms, the researcher arranged a mutually convenient date and time to interview each participant. Interviews were conducted individually, online (*n* = 10) or via the telephone (*n* = 1).

At the start of each interview, the purpose of the study was reiterated to participants and each participant was also reminded of their right to withdraw without prejudice at any time during the interview. Prior to the interview starting, participants were asked whether they had any questions and were informed that the voice recording of the interview was to begin. HAF leads were asked for their views on the aims and objectives, main considerations and challenges, and perceived impacts of the nutritional education element of HAF. On average, each interview lasted for approximately one hour. Following each interview, participants were thanked for their time and asked whether they had any questions. The voice recording was then stopped. Debrief sheets, which included researcher contact information and a unique participant number, in case participants wished to withdraw their data, were distributed to participants via email after each interview was complete. No participants opted out of the study at any stage, and all data were included in the subsequent analysis. As the interviews were audio recorded, they were then transcribed verbatim for thematic analysis.

### 2.4. Procedure for Analysis

Audio recordings of each interview were listened to in their entirety before being transcribed. In accordance with the six stages of thematic analysis developed by Braun and Clarke [22,23], transcripts were then coded and analysed. In particular, this involved reading transcripts several times to obtain familiarity with the data. Additionally, transcripts were uploaded to NVivo 12 (QSR International, Melbourne, Australia) for enhanced organisation. Using the data from the interview transcripts, codes were then identified, and subsequently collated into five main themes and numerous respective sub-themes. These themes and sub-themes are reported in the findings below.

## 3. Findings

Four main themes were identified from the narrative data collected from HAF leads: (1) the aims and objectives of HAF nutritional education; (2) the modes of delivery used for HAF nutritional education; (3) facilitators and barriers to the planning, implementation, and delivery of HAF nutritional education; and (4) the perceived impacts of HAF nutritional education. Sub-themes were also identified for each main theme, which are presented in Table 1, followed by a detailed analysis of each theme and sub-theme, and participant quotes as supporting evidence. Participant quotes were anonymised and represented by ‘P’ and a number to identify the source.

### 3.1. Theme 1: The Aims and Objectives of HAF Nutritional Education

Regardless of the mode of delivery, the aims and objectives of the nutritional education sessions were common. Two sub-themes were identified from the data collected: providing nutritional education that equips families with food-related skills and knowledge and promotes independence; signposting to relevant support in the community.

#### 3.1.1. Providing Access to Nutritional Education that Equips Families with Food-Related Skills and Knowledge and Promotes Independence

Whilst the DfE guidelines refer to HAF programmes providing education about nutrition aimed at improving children’s knowledge and awareness of healthy eating, providers delivered nutritional education in a variety of ways, and often extended provision to include parents and families:

*“We’re also trying to educate children and their wider family that actually, nutrition can be obtained in all sorts of different ways, it doesn’t have to always be through yakky things that they perhaps don’t like, like veg. Erm, that a balanced diet doesn’t have to cost the earth, and it can be accessible locally, and to be able to plan, purchase or obtain and prepare a healthy meal doesn’t necessarily have to cost the earth, and can be done within a budget and also with limited equipment. So, it’s important that we teach kids all of that stuff, but actually we are trying to sort of, teach their mums and dads as well. So that’s what we are trying to achieve out of the programme.”*

(P2)

Moreover, this included teaching families about how to make healthier versions of familiar meals:

*“So we did oven baked chicken nuggets and homemade burgers and chopped up tortillas to make your own nacho chips and stuff that were all oven baked, and people just loved it! The day was great fun making it all.”*

(P8)

To further achieve this aim, budgeting education was sometimes included within the sessions, although leads explained that this often varied in content. In some cases, financial education was provided, whereas others focused on how to make cost-effective meals, using leftovers and surplus food, and sourcing lower priced ingredients; thus, affording parents the opportunities to develop the skills required to continue supporting their children outside of the HAF programme:

*“So, how much it costs to buy a bag of carrots, you know, where to get carrots from. Erm, the fact that a bag of carrots might do two of the five meals. So actually, the financial cost of the meal is, I don’t know, five quid or whatever it comes out at, which… I mean what we are trying to do is persuade people that actually, that is far cheaper than buying three pizzas.”*

(P2)

The DfE criteria to include budgeting sessions for parents was not always so positively received, however, and was discussed by three HAF leads as a sensitive topic that should be considered with caution. One HAF lead suggested that the term ‘budgeting’ should be amended within the guidance, whilst another believed it was not suitable to cover this topic within HAF at all:

*“I don’t think it’s our place to do that. I’ve worked on the front line with communities and families who are low-income for almost my entire career, and the best budgeters I’ve ever met have been in those communities, and I don’t think the problem is budgeting, I think the problem is not having access to enough funds to buy good quality food.”*

(P1)

*“It’s a really delicate subject because it starts to challenge…you’re in a realm of conversation that can create quite a bit of stigma and worry about people and money’s always a different conversation for lots of people and certainly for those with no money, it becomes an even more difficult conversation about what you’re going to do…for me, it’s whether the word budget is the wrong word.”*

(P8)

#### 3.1.2. Signposting to Relevant Support in the Community

Alternative support was provided through signposting families to a range of relevant support services, which included community-based food aid organisations, debt advice, education services, and housing:

*“There’s a map to all of the food aid providers in the city, and then there’s also signposting to the other services. So, if it’s additional food-related support you need, or if it’s about help with your fuel and utility bills or low-income extras—all of which impacts on their food budget which then impacts on their ability to eat nutritiously. Housing benefits, employment, refugees and asylum seekers, all of that, and then links to the contact information for the organisations that can help you with that.”*

(P7)

To meet these aims and objectives, providers used various modes of delivery for nutritional education.

### 3.2. Theme 2: The Modes of Delivery Used for HAF Nutritional Education

HAF leads discussed the modes used to deliver HAF nutritional education and three sub-themes were identified: face-to-face, take-home, and online.

#### 3.2.1. Face-to-Face

Eight of the HAF leads who were interviewed explained that their nutritional education sessions were delivered face-to-face, which enabled hands-on and social activities to take place. Children, parents, and families participated in group activities such as growing and harvesting food, preparing ingredients, cooking, and eating; with staff and other families who were also involved. These sessions therefore enabled children and parents to participate in a wide range of practical food-related activities:

*“We have people like the allotment association, and they teach about growing things from seeds and the children were able to, to plant seeds which they take home and grow.”*

(P3)

The face-to-face sessions enabled families to become immersed in a sensory experience with food, where they were exposed to, and became familiar with, a range of nutritious foods in both novel and engaging ways; ranging from growing and harvesting, to tasting ingredients:

*“We also did tasting sessions, so we got children and families to try food that they wouldn’t have tried before—and the feedback from families was really surprising, such as ‘well they won’t eat that at home’, but because they prepared food and were trying it with other people, that meant that they would try it out. So, we made sure that was in there!”*

(P3)

Alongside taste-tests, families also participated in smell-based food activities, and other alternative education methods to obtain a multi-sensory food experience:

*“But at the start of the day, some of the raw ingredients were out on a table, and it was called ‘smellevision’, and the kids were just invited to come over and smell the food. They weren’t asked if they liked it or not, it was just to smell the food and smell the raw ingredients, and research shows if kids engage with the smells of raw ingredients they are far more likely to try and test different foods…it was a creative, fun, familiar but trying new things, approach to this group.”*

(P6)

*“… when they did snack time the kids got to chop up loads of fruit that they’d never had before, and they got to go along and try all the different fruit and chop it up and know what it is and name it and smell it and it was for familiarity with new fruits, veg and stuff like that.”*

(P8)

HAF leads explained that despite the coronavirus pandemic restrictions, which disrupted HAF programmes, many families still enjoyed attending face-to-face nutritional education sessions and often requested this mode of delivery:

*“we actually found that people wanted to meet face-to-face. We were very careful, kept people in family bubbles, but still managed to do the face-to-face.”*

(P3)

#### 3.2.2. Take-Home

At the end of their face-to-face sessions, some clubs ensured that families also had access to nutritious food at home, through providing take-home food parcels, including surplus food from the face-to-face sessions that they attended, meals made during the face-to-face sessions that could be cooked later at home, or stand-alone food hampers. Recipe cards often supplemented food parcels, and families were sometimes shown how to follow the recipes during the face-to-face sessions. This, therefore, reinforced the knowledge provided to families during the sessions about healthy and nutritious food, and equipped them with the skills required to prepare meals at home:

*“So we would give out any food that was left over at the end of the day and would go home with parents, so they could take it home and have it later on in the day. Everybody did a make, take and bake activity, so we knew again there was food going home. Every weekend we would give people weekend bags, which was enough food for two main meals over the weekend, so that again, we knew they were eating over the weekend. But it was about giving them the choices, giving them recipes, showing how easy it is, how you can adapt things using very few ingredients and get something that your child will eat that is nutritious.”*

(P3)

For families who could not attend face-to-face sessions at all, some providers delivered a similar but fully take-home mode of nutritional education delivery as their main method. Instead of attending a club, families collected recipe boxes, which included raw ingredients alongside recipe cards. Considerations were made surrounding the size and weight of the boxes, so that the children felt a sense of ownership and could carry them independently. Providers tried to introduce a sense of excitement surrounding the contents of the box through wrapping ingredients like a present:

*“They have been designed to be able to be carried by a child, so they are 30* *×* *20 cm^2^ and weighed no more than 2 kg, so can be carried by a young person or put in a backpack.”*

(P1)

*“So, when you open up our boxes, all of the ingredients are wrapped up in craft paper, and there is also a sticker on the box which explains what is in there and its shelf life, so when families open it we want children to feel like they are opening a present.”*

(P1)

Families could therefore prepare, cook, and eat healthy meals at home together, whilst simultaneously, but remotely, learning about food preparation and nutrition, and obtaining cooking experience:

*“They will take the take and make recipe box* *home, and when they open it they will find all the portioned ingredients to make that dish, and a recipe card—and there is also a link on the recipe card for an online tutorial video as well, and that meal can be prepared in approximately one hour with step by step instructions.”*

(P1)

#### 3.2.3. Online

Online elements were incorporated to supplement both face-to-face and take-home provision, as discussed by six of the HAF leads interviewed. For example, online tutorials were linked to recipe cards, resulting in a blended approach:

*“We have tutorial videos on YouTube and there’s a QR code on the recipe card to support families to link to those videos.”*

(P1)

In addition, one Local Authority provided families with access to an online, interactive game, which included education on nutrition and physical activity:

*“So children are collecting points for their nutrition and their activity, and then that gives them healthy hero points. So they level up to become a healthy hero, and it takes five different stages. So that is all about the big objective that they’re trying to reach. Along the whole way, they’ve got different missions that they can complete for more points, and these missions are where we then teach children about healthy living, the unhealthy habits, but they’re personified, so you might go up against the fizzy witches, and they create all these potions and powders that are affecting the townspeople, but that’s more like, in a real world, soda cans and a sugar rush. So the children learn about the effects of what the fizzy witches are doing as well as what sugar, or too much sugar, can do for your body at the same time.”*

(P4)

Importantly, the online mode of delivery lacked the hands-on cooking, sensory, and tasting experiences that the face-to-face and take-home modes provided. However, leads believed that families were still able to become familiar with ingredients and cooking skills through virtual resources and animations, which emulated aspects of real-life:

*“The biggest disadvantage that we have that we’re digital, is that you can’t really taste the numbers on the computer…but the advantage is when it comes to certain foods that they would never have seen or heard about, we can bring them to the screen. And it’s all about then saying, okay maybe we won’t be able to peel a certain vegetable or fruit that we mentioned, but you’ll know that it’s meant to be peeled, so you select peeling instead of selecting chopping. So you understand, you know, onions are chopped. And you get more about the knowledge, and then when it comes to it, whenever you have the chance, you’ll be one step ahead.”*

(P4)

Thus, whilst there were distinct modes of delivering nutritional education, some providers used more than one mode of delivery.

### 3.3. Theme 3: Facilitators and Barriers to the Planning, Implementation and Delivery of HAF Nutritional Education

Four sub-themes were identified as key factors that HAF leads took into consideration when planning, implementing and delivering nutritional education: whole-family approach; facilities at venues and at home; accessibility and inclusivity; guidelines, preferences, and quality assurance.

#### 3.3.1. Whole-Family Approach

For all modes of delivery, HAF leads explained that they considered the service user when planning, implementing, and delivering nutritional education. All HAF leads explained that their nutritional education sessions adopted a whole-family approach, which they believed to be vital in ensuring that all members of the family received well-rounded and age-appropriate support:

*“We know from research that families and children who are quite young are still aware of the food security position of their household and if they’re eating food on site at a club, but then coming home and their family are not consuming healthy and nutritious meals regularly, that still causes a lot of stress and anxiety for the young person. So in order to support families appropriately, to support children appropriately, there has to be a whole-family approach in there.”*

(P1)

*“But ultimately, is it sinking in with whole families? Because you’ve got to get a message to the parents as well. The kids can have that, but moment they then go home and then they’re getting, you know, chicken nuggets and chips because that’s what they can get from the freezer shop. Yes they’re being fed, but that’s the challenge, that’s the ultimate challenge in this as well.”*

(P8)

For example, whilst the interactive nutritional education game used by one Local Authority was primarily aimed at children, educational elements were also included for parents, coaches, and teachers who, using their knowledge, helped to support and guide children’s nutritional education, whilst being actively involved in the process themselves:

*“So the parents coaches and teachers… the children are called [Name], and then we’ve got the [Name] Academy where they all learn. And the parents, coaches and teachers, they take the role of mentors, and they are there guiding them along the whole way.”*

(P4)

Sometimes, however, it was considered appropriate to deliver entirely separate activities for children and parents. For example, education regarding signposting and budgeting was primarily aimed towards parents:

*“…the children will go off with the play workers and the parents will have a cup of coffee, and we’ll sit and talk to them about some of the issues that’s affected them. So, there is a separate session just for parents this time, because especially over the Christmas break, we feel that they need that extra support and we can give them one to one support.”*

(P3)

Consequently, ensuring that nutritional education was fun and engaging for the whole family was an important consideration for providers within all modes of delivery, so that families enjoyed participating in the sessions and therefore continued to attend:

*“It’s about actually getting them to enjoy the process of cooking and seeing it as a fun activity, rather than thinking ‘ugh I’ve got to make something, I’ve got to follow a recipe, it’s not going to work, I’m a rubbish cook’. Because that’s the feedback you get from them when they’re telling you about their cooking at home. A lot of families are really, especially mums, feel really like ‘my food is awful, they won’t eat it’ so it’s that positive reinforcement that food is actually an enjoyable thing to do together. Also encouraging an element of playfulness with food, where you feel relaxed and you feel like it’s something that is something you’d like to do again, rather than a punishment.”*

(P5)

#### 3.3.2. Facilities at Venues and at Home

The venues and facilities that were available also, in part, determined the nutritional education activities that were provided. Some providers delivered sports-related pop-up holiday clubs in parks, which therefore lacked the facilities required to prepare and cook food on-site. Instead, they included creative ways to engage children in nutritional education, such as through face-to-face discussions about nutrition and lifestyle behaviours related to sport:

*“… so you know they will be explaining, sort of, why it’s important to drink lots of fluid and not to have sugar and sugary drinks and all of that sort of stuff.”*

(P2)

However, even where clubs had on-site kitchens, they were often not big enough to accommodate face-to-face cooking sessions:

*“We have provision in really small indoor spaces that haven’t got the amenities to be able to do things like the daily cooking and various bits and pieces. So with that continuing, I don’t think you’re ever going to get the consistency around the food prep…”*

(P9)

Moreover, whilst some clubs had the necessary facilities and amenities, due to the coronavirus pandemic, they were often inaccessible as sessions were held outdoors. Providing education about preparing and cooking food without access to kitchen facilities therefore proved challenging:

*“Because it was all outside and it was quite temporary where they would put it up for the day and take it down again, we weren’t able to use electricity really or induction hobs or anything like that. And I think the next step is cooking… cooking with actual cooking facilities.”*

(P5)

Whilst this contingency method was appropriate for face-to-face nutritional education during the warmer summer months, HAF leads explained that the colder weather, such as the winter school holiday period, may create further challenges; particularly as delivering outdoor sessions may be uncomfortable and reduce the ability to provide hot food:

*“I know the case for last year was that hot food was very much a lower priority because you were eating outdoors. What I would hope is as we start to come out of COVID in future years, is that we can start putting more of a focus on the whole hot meals again.”*

(P7)

School kitchens were perceived to be the most suitable, well-equipped venues to deliver face-to-face nutritional education sessions. However, HAF leads highlighted challenges in accessing school kitchens, and explained how a lack of facilities may influence the consistency of delivery both across, and within, Local Authorities:

*“Schools weren’t particularly welcoming letting groups in to use their food tech rooms because they’re closed over the summer, so they didn’t want to open. So I think there’s some work that nationally could be done with that, actually getting schools to be more involved and say look we’ve got facilities, you’ve all got food hygiene certificates and training, come and use our kitchens—because that’s what we need, we need well kitted out indoor spaces where you can cook with ten people at once… not just one hob or a load of hobs, with an electrical system that doesn’t really work.”*

(P5)

Providers were also mindful of the facilities and amenities (e.g., cookers) available in households, as well as the cost of energy. To overcome this challenge, some providers loaned low-energy cooking equipment to families, such as slow cookers:

*“And the other thing we did is, we gave out slow cookers as well, because, especially for cheap cuts of meat, or even for vegetables, putting stuff in the slow cooker first thing in the morning… you haven’t got to think about it all day, you can go out and do all your things and then that meal is ready at night. And that was one of the things that people were saying ‘I haven’t got time to do that sort of cooking’. So it’s about quick cooking, but also the slow cooker that would enable people to fit it around their lives and still feed their children good meals.”*

(P3)

*“If you’re talking to a family where you’ve got lots of families who are living in, I don’t know, rented accommodation where they’ve all got access to one tiny kitchen with really limited kitchen cooking facilities, what’s your provision and therefore your education going to look like that’s actually going to mean anything to them? You could be presenting and providing the most useful provision with fantastic education stuff but if those families can’t actually implement that, what is the point?”*

(P7)

#### 3.3.3. Accessibility and Inclusivity

The affordability and cost of ingredients used within the nutritional education sessions was another important consideration for providers, to ensure that families could utilise skills learnt during the sessions and replicate the meals at home. Hence, many nutritional education sessions, as well as take-home recipe boxes, included basic recipes that used low-cost and easily accessible ingredients:

*“The HAF coordinator was so excited that they were doing a partnership arrangement with a supermarket, and the supermarket was providing fresh fruits and vegetables and they were encouraged to provide more exotic fruits, so the source of fruits that these kids would be seeing everyday. And on the one hand I was thinking ‘that’s fantastic it’s going to enlarge their experience and their palates and they will find all sorts of wonderful tastes’ but on the other hand I’m thinking about some really deprived communities that we’re supporting where the thought of your child coming home and saying ‘oh mum I really want more strawberries’ and you know if you can’t afford that, then that is a really horrible position to put a parent in. So I think there is something about poverty proofing that nutritional education that is really, really important.”*

(P7)

When nutritional education was delivered online, providers also considered the cost, accessibility and availability of digital infrastructure which families had at home, as well as levels of digital literacy. Importantly, HAF leads attempted to mitigate the effects of digital exclusion:

*“One of the biggest challenges was that we found the digital literacy of the parents is going to be sometimes a lot lower than their children, because the kids are more tech savvy.”*

(P4)

*“There’s quite an important reason behind that which is digital exclusion. We’re concerned that if we deliver online, then the people who need that information most are excluded from receiving it.”*

(P7)

*“Particularly in rural areas, we’ve got homes here who are off the grid. You know they’re not connected to mains electricity. They’re not connected to gas, they’ve got a generator, a diesel generator that gives them 8 h of power. So charging a device to be able to connect is just at the bottom of the list of priorities, whereas if they can travel and get to a camp that’s very different.”*

(P11)

Moreover, some families may have lacked the necessary literacy or numeracy skills required to follow recipes independently:

*“I think there’s something that isn’t taught to the Local Authorities and providers that would be a benefit, which is that they need to understand what the limitations and restrictions are that are facing the families that they are providing HAF to. By which I mean, if you’ve got an area where the majority of families’ literacy and numeracy are so poor that they’re not going to be able to follow a recipe card, then what is the point?”*

(P7)

#### 3.3.4. Guidelines, Preferences, and Quality Assurance

Inclusivity was also considered in terms of allergies, food intolerances, and culturally appropriate choices, regarding all of the food involved in the face-to-face sessions and take-home recipe boxes. Hence, recipes were often vegetarian based, which catered for many preferences, and were both sustainable and healthy choices. This was important as the boxes were widely distributed across a diverse population:

*“We did try to design the boxes and the recipes to have as few allergens as possible in the boxes and to also be suitable for as many different families as possible, so taking into account different preferences, so we wanted to widen access in that way. We also wanted the boxes to be environmentally more sustainable, and so looking at some of the recipes comparatively, that we serve in comparison to some meat-based recipes, you can see that there is a huge carbon saving by supplying vegetarian meals over meat-based meals. And also, we want our boxes to be very healthy, to meet School Food Standards, and to support children with creating and producing high quality, high nutritious value meals in their home, and we felt that was best done through vegetarian food that was based on pulses, and fresh fruits and vegetables.”*

(P1)

Clear, mandatory guidelines were outlined by the DfE, which stipulated that all food prepared and consumed, and all food-related activities, must conform to School Food Standards:

*“So as part of the grant agreement, the Food Standards are linked in there and they’re signed up to having to follow the School Food Standards as part of that.”*

(P7)

*“We did lots of mandatory training around, kind of, food hygiene and achieving School Food Standards, menu development and allergy awareness, etc. So almost kind of the compliance end of the spectrum on the food side was where we spent quite a lot of our energy.”*

(P9)

Spot checks were often used to determine whether School Food Standards were met:

*“But at the clubs where they either prepare or consume food on site, one of the things that we put in place this year was spot checks through our school meals team, to actually go in and look at the type of food that was being offered, the standard and value of that food, and to make sure that there was some educational support that was associated with it.”*

(P2)

Moreover, HAF leads were aware of health and safety protocols and guidelines, which were also important considerations set out by the DfE:

*“The guidelines that we follow are health and safety. So clearly, the pantries are registered with environmental health, and the food that we give out is part of that. So, we would only give out food that’s fresh, and we link with our food banks as well so that we make sure that bags that we are giving out are covered by that.”*

(P3)

Importantly however, whilst clear guidelines were stipulated regarding food provision and health and safety, HAF leads explained that no clear guidance was outlined by the DfE regarding the content of nutritional education, and highlighted the challenges that arose due to the vagueness of this information. Instead, some providers referred to the national curriculum and NHS guidance when planning, implementing, and delivering nutritional education:

*“In terms of guidelines for the home element, in terms of what should be taught, not really... it’s more of a case of looking at what’s on the market, you know, things like what’s ‘Change4Life’ doing, and if ‘Change4Life’ is coming off from the NHS and public health, then surely they’ve done enough research into why they’ve taught these subjects. So that formed the basis there, and then it came to looking at the school national curriculum.”*

(P4)

*“No, it says in the guidance it’s to provide enriching activities to help with skills and confidence and things like that, and we want to provide nutritional education to help families understand more about eating healthily, cooking on a budget, etc. It’s something around those lines, so there’s no actual guidance.”*

(P8)

Hence, many providers delivered nutritional education informally, weaving this element of HAF into other activities:

*“I guess it’s what you class as nutritional education, because some of them will be running nutritional education by stealth. So, for some of these groups it will be a really big win if these kids aren’t eating chicken and chips seven days a week, and that they are now eating some pasta with a bit of vegetables in it, or a sandwich and some carrots and apples and grapes as well. You know, that is a huge win for some of these groups. So, it really depends on what you class as nutritional education. If you’re talking about formal education, I would say not all of them do it because loads of them will be building it into their activities, because otherwise why would their children and their parents choose to come along to that? It won’t happen.”*

(P7)

As such, some Local Authorities and organisations created their own quality assurance protocols for HAF nutritional education. This included spot checks and in-house training:

*“We don’t just let them do it. So, somebody goes in on a regular basis. There’s a coordinator for each of the areas, and they go in and they report back and send photos on what’s happening. So, it’s a monitoring system…”*

(P3)

However, other HAF leads explained that nutritional education was not a main priority in the delivery of their HAF programme in 2021, with seven of the HAF leads referring to the vagueness of the DfE’s guidelines on nutritional education, alongside the complexity of implementing this element of the HAF programme within a short time-frame:

*“I think it’s been well recognised from the outset, I think from an operational delivery point of view, it’s been something that if I’m really honest, we’ve been conscious of, but its probably took us some time to really get our heads around in terms of how you deliver a really great programme.”*

(P2)

*“I think we can be really honest now in our reflection of the nutritional education side of the programme, that it was quite weak because we didn’t put emphasis on it. I think we have to be really honest and not challenging our providers too much because we didn’t put the emphasis on that as an outcome that we were looking for them to achieve. I think probably even when we consider the guidance document that was shared with providers ahead of HAF grant applications, there wasn’t a huge focus on nutritional education and associated outcomes. It was very much around the School Food Standards side of the programme.”*

(P9)

### 3.4. Theme 4: The Perceived Impacts of HAF Nutritional Education

Three sub-themes were identified regarding the perceived impacts of nutritional education: improved cooking confidence and competence; enhanced willingness to try new foods; and, better understanding of sustainability, environmental impacts, and food provenance.

#### 3.4.1. Improved Cooking Confidence and Competence

The practical experiences involved in face-to-face nutritional education sessions and within the take-home recipe boxes, such as preparing ingredients and cooking, were perceived to improve the confidence and competence of families regarding their cooking ability:

*“They were more relaxed I think and more relaxed around lunch time and more confident after the sessions, you know…”*

(P6)

*“Some of this was about cooking with confidence, so a lot of the families that didn’t have the confidence to cook or to step outside of their usual group of meals that they would cook. So it was about providing them with the recipe cards and all of the resources that would enable them to try and cook those things, but encouraging them to do that, if time allowed, as a family, so it became a family activity.”*

(P7)

Through participation in nutritional education, families became familiar with novel ingredients and kitchen equipment, and gained the skills required for food preparation. HAF leads perceived this as a beneficial method of improving families’ competence and confidence in preparing and tasting novel foods, which were transferrable skills they could use in their everyday lives:

*“But having the parents and sometimes the grandparents there, was key—because if kids come home with a bag of food, parents will say, well if they’re not used to cooking from scratch, they’ll say ‘what am I going to do with that?’. But instead, in this case, at lunchtime they had this delicious meal and I was showing them how to make it, so it was less foreign.”*

(P6)

#### 3.4.2. Enhanced Willingness to Try New Foods

Nutritional education sessions additionally allowed for a hands-on, practical experience with food preparation and cooking within a low-risk environment that supported children and parents to try novel foods. HAF leads were mindful that for many families, involving children in the kitchen and cooking unfamiliar meals at home is often a risk, due to possible food waste and spoilage which they cannot afford, thus potentially limiting the range of foods they have tried. HAF leads stated that the nutritional education sessions, which provide families an opportunity to experience and practice skills without monetary risk, empowered children and parents to engage in the sessions, and afforded families food-related experiences, which they may otherwise not have encountered:

*“I know from my experiences of working with families for many years who are at risk of, or in food insecurity, that sometimes in those households parents don’t feel confident having their children in the kitchen because of the risk of spoilage, waste, all sorts of issues related basically to the loss of money. And so as cooking is deemed a fairly high risk activity, it can be those children from families who are in food insecurity who miss out on that. So this offers the families, and the children, an opportunity to do that together risk-free, or risk-free in a monetary sense, and it also offers those families who, again may not have very much money to buy new foods and try it, again that offers no risks through wastage and spoilage, and it gives them an opportunity to try recipes and try new foods that they wouldn’t have otherwise, risk-free from a monetary perspective.”*

(P1)

*“So at home, if you are living with food insecurity and you don’t have disposable income to spare, you’re not likely to go out and buy a load of random ingredients you haven’t had before, just in case the child doesn’t like it or it gets wasted. So we try to make our sessions as open as possible, so that they can have that experience. So in term-time that’s in nurseries, but during the HAF it was during the food sessions.”*

(P5)

HAF leads perceived a positive shift in children’s willingness to try new foods through attendance at the sessions. In addition to the financially low-risk environment, leads suggested that vicarious learning, through observing and modelling peers during the sessions, may have also played a role in influencing these changes:

*“They would eat with their peers, they would see what their peers were eating and you could provide more positive reinforcement to them to be eating healthy meals and trying things that they haven’t tried before, and changing their eating habits. And we would always have parents and carers saying ‘Oh I’ve tried to get them to eat this stuff at home they won’t, it’s great now they’re coming home asking for it’ so that’s always great to hear.”*

(P7)

#### 3.4.3. Better Understanding of Sustainability, Environmental Impacts, and Food Provenance

Leads believed that sessions that involved growing and harvesting increased families’ awareness of food sustainability, environmental impacts, and food provenance, and perceived this to play a role in influencing children’s willingness to try new foods:

*“We also have a number of community organisations, more each year, that are actually growing foods and then the children are involved in growing that food, harvesting that food, preparing that food and then eating that food. And again, I think that’s a really important way, particularly in terms of fresh fruit and vegetables, of getting children and young people really engaged and interested in eating fresh fruit and vegetables that they ordinarily wouldn’t eat.”*

(P7)

Similarly, including information about sustainability, environmental impacts, and food provenance on cards, leaflets, or in group discussions allowed families to contextualise their food and educated them about environmentally friendly and sustainable ingredients, which are both healthy and nutritious:

*“So, the reason that we include environmental information on the cards is because we want to give young people and their families an opportunity to contextualise the food that they are eating, and indeed any food that they are eating, and we think it’s a really good opportunity to support young people to understand a little bit more about the links between food and the environment.”*

(P1)

## 4. Discussion

Whilst the DfE quality standards framework broadly outlines the mandatory inclusion of nutritional education in HAF programmes delivered across all 151 higher-tier Local Authorities in England, to the authors’ knowledge, no research has yet explored this element of HAF. This study, therefore, explored the implementation, delivery, perceived facilitators, barriers, and impacts of HAF nutritional education through qualitative interviews with 11 HAF leads from across eight Local Authorities in England. In this section, we will firstly present the overall findings regarding modes of nutritional education delivery, highlight key themes with reference to existing literature, and suggest practical considerations for both implementation and delivery.

Overall, the findings of the current study demonstrate that nutritional education (as defined by the DfE’s HAF guidance) is not delivered in a uniform manner, both across and within Local Authorities, with three main modes of delivery identified: face-to-face, take-home, and online. Local flexibility may be an advantage as it provides flexibility in delivery to utilise local assets (e.g., staff expertise, venues) and empower local community organisations, within a particular context, such as during the coronavirus pandemic. However, it may also lead to patchy coverage, especially in areas where there is a lack of facilities and staff expertise. Moreover, HAF leads highlighted the coronavirus pandemic as a potential challenge for the delivery of face-to-face cooking sessions through these contingency methods in colder weather, and outlined concerns regarding their adherence to health and safety guidance if these restrictions remained a barrier to using indoor facilities in future.

However, the DfE website suggests that nutritional education sessions should be delivered face-to-face at HAF programmes, and numerous research studies lend support to hands-on, practical involvement in food preparation and cooking [16,24]. Furthermore, there is a paucity of peer-reviewed research supporting the effectiveness of take-home and online delivery methods against outcomes such as cooking competence and the willingness to try new foods. For all modes of delivery, the overarching aims of nutritional education were to equip families with skills and knowledge that promote food-related independence, to signpost families to relevant support services, and to provide food and lifestyle related education in a fun and engaging way. Indeed, HAF leads explained that they delivered more than simply ‘nutritional’ education, and instead involved a range of food-related information, to improve families’ overall food literacy. This is promising as prior research has shown that school-based nutritional education interventions, which similarly demonstrate a whole-school approach are beneficial for improving a range of outcomes, including wellbeing, dietary intake, and cooking competence and confidence [18]. Moreover, the fact that providers considered food literacy to be additional to nutritional education suggests that some providers may have produced their own working definition of what they consider nutritional education.

Many HAF leads highlighted a preference for face-to-face nutritional education that provides supervised access to nutritious food on-site and facilitates hands-on, experiential learning, such as growing and harvesting ingredients, as well as preparing and cooking meals, in a social context. Often, families participated in these experiences together, as well as alongside holiday club staff and other families who also attended. Importantly, face-to-face sessions additionally allowed for families to become immersed in multi-sensory experiences with food, including tasting, smelling, and touching often unfamiliar ingredients, which prior research has demonstrated to be successful in other contexts [25,26]. Multi-sensory exposure has been found to increase children’s willingness to try and taste vegetables [27], and similarly, HAF leads highlighted that these multi-sensory sessions were extremely engaging, and perceived participation to enhance families’ willingness to try the foods involved. Thus, incorporating sensory education may be an important consideration for all providers in future HAF delivery.

Supplementary to the face-to-face sessions, some programmes delivered nutritional education online. Websites, which included resources such as recipes, tips, and online cooking tutorials were used by some providers to facilitate the educational element of take-home recipe boxes and food parcels. Others utilised online delivery by involving families in food and lifestyle related games, which have been shown to enhance engagement and improve nutritional knowledge in other food-related contexts [28]. Despite the perceived benefits of nutritional education, however, digital literacy and digital exclusion should be considered by providers if any element of nutritional education within HAF is delivered online. The coronavirus pandemic has widened the already prevalent digital divide in the UK, where the likelihood of having at-home internet access increases with higher income [29]. Especially if the coronavirus pandemic results in hybrid provision in the future, where some activities may be face-to-face and others delivered online, this is an important consideration to ensure that all families can access the provision that they require.

Furthermore, this remote method of education omitted hands-on practical food experiences, and further research is therefore required to investigate the efficacy of this method against a number of outcome measures, such as dietary behaviour change and health literacy. Nevertheless, HAF leads perceived participation in all modes of nutritional education delivery as beneficial in increasing families’ familiarity with novel ingredients and skills, and subsequently improving dietary habits, cooking competence, and confidence. Indeed, prior research has similarly found an association between exposure and consumption, and that even mere exposure to unfamiliar target foods can increase the willingness to try them [30], supporting the perceived impacts of participation in the sessions, as highlighted by HAF leads. Moreover, previous school-based research has similarly demonstrated improved dietary intake, and cooking competence and confidence following attendance at chef-led cooking interventions [16,31], which are similar modes of delivery as those used within the HAF face-to-face and online cooking tutorials.

Although take-home and remote modes of delivery omit the social element of face-to-face sessions with peers, family members may still participate with one another within the home. Due to the financial risk involved in wasting or spoiling ingredients when cooking with children who are inexperienced, this social activity may otherwise not be possible for many families facing household food insecurity. As such, HAF leads explained that providers often further facilitate the social element of HAF by providing a whole family and whole community approach to nutritional education, often extending provision to allow families who are not in receipt of free school meals during term-time to participate; thus affording families food-related social experiences, which they otherwise may not encounter. Moreover, leads explained the importance of a whole-family approach as a way of engaging with the household budget holder and to support decision makers in shopping for nutritious food; whilst upskilling parents to prepare and cook a wide range of healthy but affordable meals. Including information about food provenance, sustainability, and environmental impacts may provide a further tool to engage young people in sessions and may lead to positive impacts beyond dietary behaviours. Indeed, psychological theory demonstrates that enhanced knowledge of a phenomenon can result in changed behaviour, and the family system has an active role in influencing children’s eating habits [32]. Through involvement in whole-family education sessions, all family members can subsequently obtain an awareness of any necessary food-related behaviour changes for a healthy lifestyle, as well as an understanding of issues such as sustainability and environmental impacts. Additionally, based on Evaluative Conditioning Theory [33,34], the social element of participating in nutritional education with peers or family can create a positive association with food, alongside facilitating vicarious learning, and has previously been found to improve dietary habits and a range of psychosocial factors, including cooking confidence and self-efficacy, for both the children and parents involved [34]. Hence, adopting a whole-family approach may be an important consideration for the implementation and delivery of these sessions at the local level.

Further considerations are required regarding the facilities and skills that families can access at home, especially where the level of support and resources provided at face-to-face HAF clubs is unavailable [35]. Individual, household, and community factors such as health literacy, language skills, household cooking facilities, food deserts, and household income should not result in families being excluded from accessing the full range of HAF nutritional education resources. For example, families may be unable to afford to buy ingredients, or lack access to kitchen equipment and facilities at home that were used during face-to-face sessions. Indeed, leads highlighted that whilst providing novel ingredients for nutritional education may be engaging for the families, the longevity of the perceived influence of this inclusion may be limited if the ingredients are not affordable for households. Hence, poverty proofing provision, through considering these implications in the planning, implementation, and delivery of nutritional education is vital for both the efficacy and sustainability of this part of the HAF programme. HAF providers should facilitate optimum standards of nutritional education that all families, regardless of background, may continue to use in their lives outside of HAF.

Similarly, Local Authorities should consider the venues and facilities available to deliver face-to-face sessions, with school kitchens depicted by HAF leads as the most suitable venue for delivering nutritional education during school holiday periods. However, leads also highlighted the challenges that often arise when trying to secure these facilities. Although there are many examples of innovative practice, such as cooking outside on portable stoves, making fruit smoothies, community sports pop-ups and preparing cold meals, these are unlikely to be long-term solutions to this issue, and may have implications for the consistency and standard of nutritional education at HAF programmes across England.

Importantly, leads highlighted a lack of DfE guidance surrounding the content for nutritional education with many leads expressing concerns surrounding consistency across and within Local Authorities. Hence, some Local Authorities implemented their own quality assurance procedures to manage this process at a local level. Whilst HAF leads reported that families used the take-home food boxes to participate in home cooking activities, further evaluation is required to investigate whether adults, and children, use these boxes for these activities, what learning occurs, and other further impacts (e.g., improved dietary intake). Possible evaluations could use digital ethnography or self-reported questionnaires. Despite these challenges, all HAF leads were enthusiastic and about providing nutritional education to children and families and displayed overarching positivity regarding this element of the programme. Based on knowledge of the perceived impacts that the nutritional education element of HAF offers, we suggest that the DfE considers conducting a process and impact evaluation of the different modes of delivery to determine which are most effective, under which contexts, and why. These findings would enable the DfE to provide more detailed guidance to Local Authorities on the nutritional education element of HAF within the quality standards framework. Furthermore, we suggest that local monitoring of implementation and delivery is required, to support improved service provision, to ensure daily provision, and shared learning across Local Authorities and holiday clubs.

## 5. Conclusions

The current study provides novel insight into the various modes used for the implementation and delivery of nutritional education by Local Authorities, as well as highlighting the perceived facilitators, barriers, and impacts of this element of HAF. A limitation of the current study is that we purposively sampled HAF leads from across eight Local Authorities, and this may not provide a representative picture of nutritional education across England. By sampling through the HAF Alliance, leads had vast experience and involvement with holiday clubs, or HAF through the pilot phase. Whilst the HAF leads interviewed did not highlight challenges surrounding family engagement and uptake of the nutritional education sessions, this may not necessarily be the case for Local Authorities with less experience. Nevertheless, the findings of the current study raise numerous important issues relating to policy and practice of delivering nutritional education within the HAF programme. To the authors’ knowledge, this is the first study to explore this mandatory element of HAF. Given sampling limitations, we suggest that future research aims to obtain a national picture of how nutritional education is implemented and delivered across the HAF programme in England, which modes of delivery prove most effective against numerous outcome measures, and importantly, how nutritional education is delivered by holiday clubs themselves. The qualitative findings presented should help guide the focus of any such research efforts.

## Figures and Tables

**Table 1:** Themes and sub-themes identified through interviews with HAF leads in England.

Theme	Sub-Themes
The aims and objectives of HAF nutritional education	Providing nutritional education that equips families with food-related skills and knowledge and promotes independence; signposting to relevant support in the community.
The modes of delivery used for HAF nutritional education	Face-to-face; take-home; online.
Facilitators and barriers to the planning, implementation, and delivery of HAF nutritional education	Whole-family approach; facilities at venues and at home; accessibility and inclusivity; guidelines, preferences and quality assurance.
The perceived impacts of HAF nutritional education	Improved cooking confidence and competence; enhanced willingness to try new foods; better understanding of sustainability, environmental impacts and food provenance.

## Data Availability

To request access to the data, please contact the corresponding author. The data are not publicly available to prevent identification of participants.
